# Intravenous immunoglobulin as an important adjunct in the prevention and therapy of coronavirus 2019 disease

**DOI:** 10.1111/sji.13101

**Published:** 2021-09-16

**Authors:** Maria Giovanna Danieli, Mario Andrea Piga, Alberto Paladini, Eleonora Longhi, Cristina Mezzanotte, Gianluca Moroncini, Yehuda Shoenfeld

**Affiliations:** ^1^ Clinica Medica, Dipartimento di Medicina Interna, AOU Ospedali Riuniti di Ancona and DISCLIMO Università Politecnica delle Marche, Clinica Medica Ancona Italy; ^2^ School of Specialisation in Allergology and Clinical Immunology, Dipartimento di Medicina Interna, AOU Ospedali Riuniti di Ancona Università Politecnica delle Marche Ancona Italy; ^3^ School of Specialisation in Internal Medicine, Dipartimento di Medicina Interna, AOU Ospedali Riuniti di Ancona Università Politecnica delle Marche Ancona Italy; ^4^ Scuola di Medicina e Chirurgia Alma Mater Studiorum Università degli Studi di Bologna Bologna Italy; ^5^ Ariel University Ariel Israel; ^6^ The Zabludowicz Center for Autoimmune Diseases Sheba Medical Center Ramat Gan Israel; ^7^ Saint Petersburg State University St. Petersburg Russia; ^8^ I.M. Sechenov First Moscow State Medical University of the Ministry of Health of the Russian Federation (Sechenov University) Moscow Russia

**Keywords:** autoimmunity, Coronavirus disease‐19, COVID‐19, COVID‐19 vaccination, immunomodulation, intravenous immunoglobulin, Long‐COVID, Post‐COVID

## Abstract

The coronavirus disease‐19 (COVID‐19) pandemic caused by severe acute respiratory syndrome coronavirus 2 (SARS‐CoV‐2) challenged globally with its morbidity and mortality. A small percentage of affected patients (20%) progress into the second stage of the disease clinically presenting with severe or fatal involvement of lung, heart and vascular system, all contributing to multiple‐organ failure. The so‐called ‘cytokines storm’ is considered the pathogenic basis of severe disease and it is a target for treatment with corticosteroids, immunotherapies and intravenous immunoglobulin (IVIg). We provide an overview of the role of IVIg in the therapy of adult patients with COVID‐19 disease. After discussing the possible underlying mechanisms of IVIg immunomodulation in COVID‐19 disease, we review the studies in which IVIg was employed. Considering the latest evidence that show a link between new coronavirus and autoimmunity, we also discuss the use of IVIg in COVID‐19 and anti‐SARS‐CoV‐2 vaccination related autoimmune diseases and the post‐COVID‐19 syndrome. The benefit of high‐dose IVIg is evident in almost all studies with a rapid response, a reduction in mortality and improved pulmonary function in critically ill COVID‐19 patients. It seems that an early administration of IVIg is crucial for a successful outcome. Studies’ limitations are represented by the small number of patients, the lack of control groups in some and the heterogeneity of included patients. IVIg treatment can reduce the stay in ICU and the demand for mechanical ventilation, thus contributing to attenuate the burden of the disease.

**Abbreviations**
ADCC: antibody‐dependent cytotoxicity
COVID‐19: coronavirus disease‐19
IL: interleukin
IVIg: intravenous immunoglobulin
SARS‐CoV‐2: severe acute respiratory syndrome coronavirus 2

## 1 INTRODUCTION

The recent coronavirus disease‐19 (COVID‐19) pandemic challenged globally with its morbidity and mortality.^1^, ^2^ In most of the affected patients, the severe acute respiratory syndrome coronavirus 2 (SARS‐CoV‐2) infection is characterized by mild symptoms linked to the viral replication in the upper respiratory tract. A small percentage of patients (20%) progress into the second stage of the disease, clinically presenting with severe or fatal involvement of lung, heart and vascular system, all contributing to a multiple‐organ failure. Currently, few clinical or biological markers have been identified which may help in predicting the course of the disease.^2^, ^3^, ^4^, ^5^, ^6^, ^7^ Several biohumoral mediators have been postulated, such as ferritin, pro‐inflammatory cytokines (interleukin‐1 [IL‐1] and IL‐6) and chemokines (IL‐8).^8^, ^9^ Even with the current understanding of new pathogenic mechanisms, questions remain what the specific pharmacological objective is. As the mechanisms might suggest, one of the strongest targets for therapy could be aberrant immune response and cytokine‐mediated inflammation, manageable with corticosteroids, passive immunization, immunotherapies – such as neutralizing antibodies to IL‐6 and recombinant IL‐1 receptor antagonist – convalescent plasma and intravenous immunoglobulin (IVIg).^10^, ^11^, ^12^

Although immunosuppression might be a suggested solution, immunomodulation through IVIg offers a lower exposure to adverse effects and a safer therapeutic mechanism.

The therapeutic benefits of IVIg could be linked to the anti‐inflammatory and immunomodulatory properties.^13^, ^14^ In autoimmune neurological diseases, such as Guillain‐Barré syndrome, chronic inflammatory demyelinating polyneuropathy, and multifocal motor neuropathy, IVIg is positively employed as first‐line therapies.^15^

In COVID‐19, these immunotherapies, through different mechanisms, recognize three principal targets: control of cytokine storm, viral neutralization and restoration of immune dysregulation.^16^, ^17^ As in autoimmune and (auto)inflammatory diseases, even in COVID‐19, multiple research reports documented several distinct humoral and cellular anomalies and cytokines imbalance. An effective treatment must impact these multiple targets. In this sense, IVIg is a good candidate, since IVIg can act at several levels with different and synergic mechanisms, thus restoring the immune system homeostasis.^13^, ^16^ Further development of IVIg therapy is linked to the development of specific monoclonal antibodies (MAbs). There are now several clinical studies evaluating specific MAbs against SARS‐CoV‐2, as recently reviewed in Kumar et al.^14^

With this review, we point to the role of IVIg in the therapy of adult patients with COVID‐19 disease.

## 2 METHODOLOGY

This review has been drafted following the main rules of scientific narrative writing to respect the objectivity of collected data. On 8 May 2021, we have performed a comprehensive coverage of literature focused on topic ‘COVID‐19’ and ‘IVIg’, published from 2019 on main online libraries and databases, such as MEDLINE/PubMed, Scopus, Web of science and LitCovid. The search was repeated monthly until August 2021.

We have considered sources with the highest level of evidence, citing online journals when reporting significant clinical data to ensure the greatest possible updating.

Once all bibliographic items were collected, we have critically analysed and then organized the information by describing the main results to draw general conclusions, summarizing new evidence‐based points.

## 3 IMMUNOLOGICAL EFFECTS OF IVIG IN COVID‐19

**FIGURE 1:**
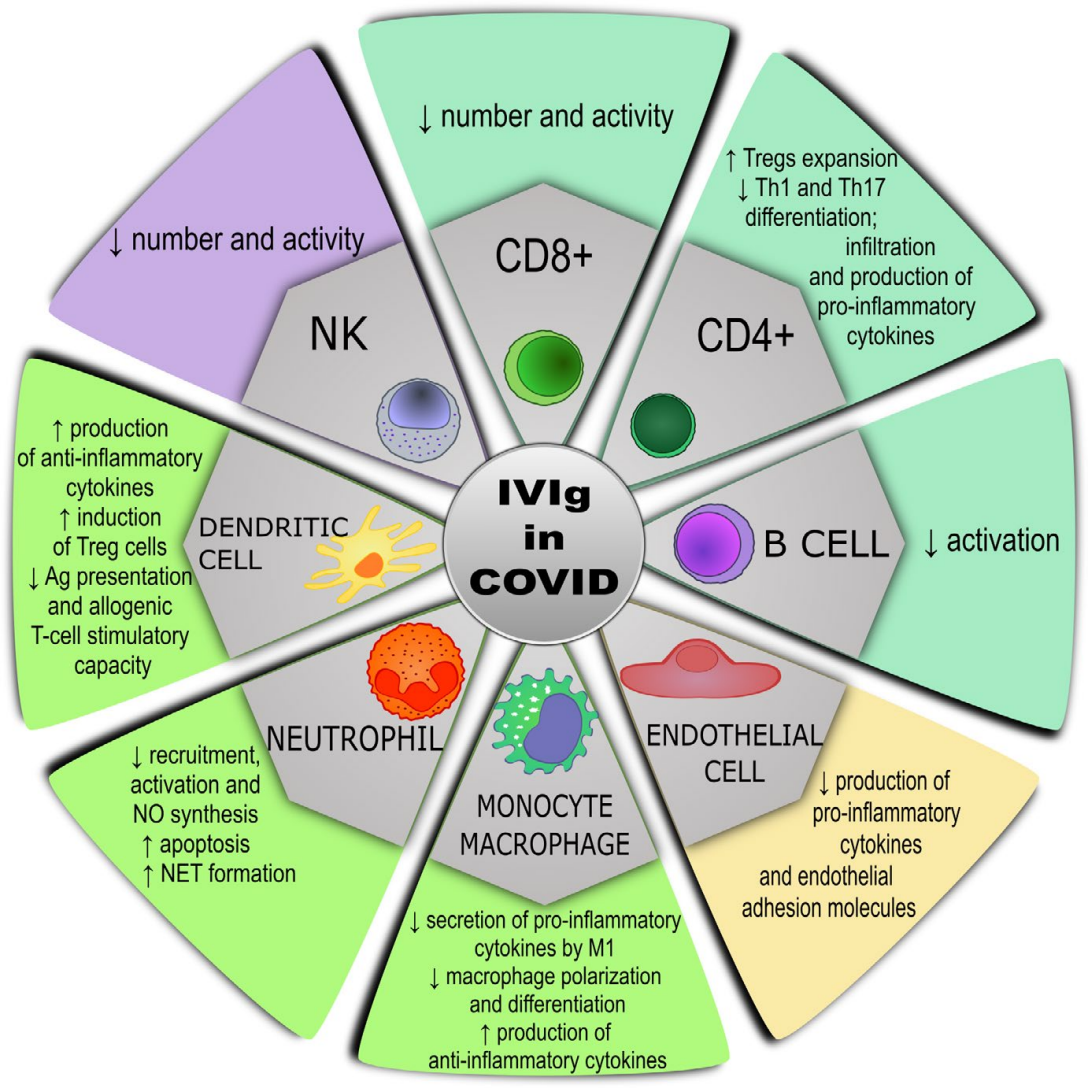
Proposed mechanisms of action of IVIg in COVID‐19 infection

Several mechanisms have been reported to explain the beneficial effect of IVIg in regulating the immune response and in treating viral infections, including SARS‐CoV‐2. These comprise autoantibodies neutralization, modifications of cytokine production (pro‐inflammatory vs anti‐inflammatory), inhibition of complement activation, killing of target cells by antibody‐dependent cytotoxicity (ADCC), and control of cell‐cell interaction through the blockade of Fc gamma receptors on immune cells (Figure 1).^14^, ^15^, ^16^ The cytokines storm is an essential feature of COVID‐19 and is characterized by a high expression of IL‐6 and tumour necrosis factor [TNF]‐α.^18^, ^19^, ^20^, ^21^, ^22^ Basically, IVIg treatment mainly interfering at cellular and humoral level can impact the subsequent production of inflammatory biologically active molecules.^18^, ^19^

IVIg can act on various types of immune cells involved in SARS‐CoV‐2 infection (Figure 1).

### 3.1 CD4+ T lymphocytes

An increased release of pro‐inflammatory cytokines by Th1 and Th17 cells (eg IL‐6 and IL‐17) was documented in COVID‐19 patients, associated with the inflammatory condition.^23^, ^24^, ^25^ High‐dose IVIg has been shown to inhibit the activation and production of cytokines by Th1 and Th17 cells, and further restore the balance between Th1, Th2, Th17 and Treg cells.^24^, ^25^, ^26^ In vitro and in vivo studies reported that high‐dose IVIg favours the expansion of Tregs, modulating their activity which involves through various mechanisms the dendritic cells (DCs), including internalization and presentation of Treg epitope peptides content in IVIg.^27^, ^28^

### 3.2 CD8+ T lymphocytes

CD8+ T lymphocytes are involved at different levels in viral infections. In COVID‐19, the release of cytokines and cytotoxic granules by activated CD8+ cells can contribute to and aggravate cytokines storm. In human and in experimental models of autoimmune diseases, high‐dose IVIg causes a decrease in CD8+ T cells, through specific antibodies directed to T cells or *via* interactions between antigen presenting cells (APC) and T‐cell receptor (TCR) signalling.^29^

### 3.3 B lymphocytes

High‐dose IVIg inhibits activation of B cells; an important mechanism in this process is the neutralization of BAFF and APRIL (members of tumour necrosis factor family) by IVIg.^30^ Another mechanism is the interaction between the sialylated Fc fragment of IgG and CD22 with subsequent promotion of B cell apoptosis^31^; IVIg also inhibits Toll‐like receptor 9 (TLR‐9) pathway, down streaming in this way the production of cytokines by B cells.^32^ Yet no data are available on IVIg action on SARS‐CoV‐2‐specific memory B cells which are generated during the acute phase of COVID‐19.^33^

### 3.4 Endothelial cells

SARS‐CoV‐2 infection could lead to endothelial damage through complement activation thus triggering the procoagulant state. Besides its role in interfering complement pathways, i*n vitro* studies shown that high‐dose IgG inhibits the production of pro‐inflammatory cytokines (eg IL‐6, G‐CSF and IL‐1β) in cultured human coronary artery endothelial cells.^34^

### 3.5 Monocyte‐macrophages

in vitro, IVIg inhibits the secretion of pro‐inflammatory cytokines, such as IL‐6, by M1 macrophages and elicits macrophage polarization; in addition, IVIg restricts the differentiation of macrophages and enhances the production of anti‐inflammatory cytokines, as IL‐10 by LPS‐stimulated monocytes.^35^ Other interesting data come from the studies of Saha et al^36^ on the polarization of macrophages which showed that IVIg can suppress the activity of both classic pro‐inflammatory M1 and anti‐inflammatory alternative M2 macrophages.

### 3.6 Dendritic cells

High‐dose IVIg inhibits these cells activation and stimulates the production of anti‐inflammatory cytokines by immature DCs; moreover, IVIg might favour the induction of Treg cells by DCs and suppress antigen presentation and allogenic T‐cell stimulatory capacity.^37^ In a recent series of experiments, Karman et al^38^ explored the role of IVIg on β‐catenin, crucial component in the Wnt pathway and, subsequently, on the control of DCs. Even if IVIg can interact with β‐catenin, this seems not essential for the anti‐inflammatory properties of IVIg.

### 3.7 Neutrophils‐granulocytes

In severe COVID‐19 disease, an increase in CD62L(dim) neutrophils can contribute to pulmonary embolisms.^39^ The role of IVIg is complex and relates to inhibition of recruitment, activation and synthesis of nitric oxide by neutrophils. IVIg can reduce the production of neutrophil extracellular traps which are in part responsible for cytokines storm and organ damage in COVID‐19.^40^ Anti‐Siglec autoantibodies (antibodies to sialic acid‐binding immunoglobulin‐like lectin) present in IVIg preparations can stimulate PMN apoptosis.^41^

### 3.8 Natural killer cells

We did not find any specific data on the impact of IVIg on NK cells in SARS‐CoV‐2 infection. In patients treated for their autoimmune diseases, high‐dose IVIg causes a decrease in number and activity of NK cells, however not affecting their properties for controlling viral infections and malignancies.^42^ In vivo NK cells can secrete IFN‐γ and TNF‐α with immunoregulatory action that help in controlling viral infections. In COVID‐19 NK cells can contribute to control viral infections through viral clearance and modulation of cytokine storm.^43^ Patients with severe COVID‐19 have a significant reduction in NK cells as compared to patients with mild SARS‐CoV‐2 infection.^44^

Besides immune system cells, even specific antibodies can contribute to the severity of the disease in SARS‐CoV‐2 infections. Some studies investigate the hypothesis that qualitative modifications in IgG structures of anti‐SARS‐CoV‐2 antibodies can promote the inflammatory response and thus contribute to the worsening of the disease. Hoepel et al^45^ documented that sera from critically ill patients contain high titres of specific SARS‐CoV‐2 anti‐spike protein IgG (anti‐S IgG). These anti‐spike IgG antibodies have pro‐inflammatory properties due to different glycosylation, mainly low fucosylation of the Fc fragment. Afucosylated IgG have been detected in sera from critical patients and not from mild disease. As demonstrated by Larsen et al,^46^ afucosylated IgG in vitro stimulates the release from macrophages of IL‐6, one of the key cytokines in COVID‐19. Moreover, afucosylated IgG is a strong immune response, usually directed against alloantigens on blood cells and enveloped viral‐infected cells. Whereas this response could be beneficial in some viral infections, as HIV, it could enhance the severity of COVID‐19 in certain conditions.^46^ We do not know if IVIg can interfere or modify this response through the modulation of Fcγ receptors. However, since the anti‐S specific IgG response with low core fucosylation take place around 1‐2 weeks after onset of symptoms, this could explain the major benefit of IVIg if administered in the early phase of the disease.

The role of SARS‐CoV‐2‐ specific neutralizing antibodies is complex. Higher titres of these antibodies are associated with higher COVID‐19 diseases’ severity. In a series of experiments, Adeniji et al^47^ demonstrated that SARS‐CoV‐2‐specific neutralizing antibodies from hospitalized patients (ie with a severe disease) through their Fc receptors prompted higher antibody‐dependent complement deposition (ADCD) and lower antibody‐dependent cell‐mediated phagocytosis (ADCP) compared to antibodies from non‐hospitalized patients (ie with mild disease). Since higher ADCD and higher ADCP are associated with higher and lower systemic inflammation during COVID‐19, respectively, Authors^47^ postulated that qualitative, and not only quantitative, differential features of anti‐SARS‐CoV‐2 antibodies can influence the severity of the disease. It is important to understand how these antibodies or antibodies contained in IVIg preparations, interplay between them and thus can modify disease's severity.^47^

Another interesting mechanism is that related to antibody‐dependent enhancement (ADE) in SARS‐CoV‐2 infections.^48^ ADE is a phenomenon where pre‐existing poor neutralizing antibodies facilitate viral access via FcγRs leading to enhanced infection or immune system imbalance. In SARS‐CoV‐2 infection, antibodies derive from cross‐reaction against other coronaviruses serotypes. The potential role of high‐dose IVIg for inhibiting ADE phenomenon might lie in the saturation of activating FcγRs and FcRn and subsequent reduction of immune complexes access and enhancement of impaired antibody clearance.^48^

Besides their main content in IgG, there are some IgA‐enriched or IgM enriched preparations of IVIg. Some evidence highlighted the specific role of these two classes of immunoglobulin in the modulation of the immune response. Saha et al^49^ documented that monomeric IgA present in IVIg preparation can inhibit differentiation and amplification of human Th17 cells and their release of IL‐17A. In another studies, IgM‐enriched immunoglobulins (Pentaglobin) has been shown to improve the microcirculation and the anti‐inflammatory response in sepsis and septic shock.^50^, ^51^ However, no data are now available about IgA‐ and/or IgM‐enriched IVIg preparations as possible treatment of COVID‐19 hyperinflammation.

## 4 IVIG CONTAINS ANTI‐SARS‐COV‐2 ANTIBODIES

Recent papers reported that some available IVIg preparations contain antibodies that react against SARS‐CoV‐2 antigens in vitro. Diez et al^52^ found significant cross‐reactivity with SARS‐CoV‐2, MERS‐CoV, and other coronaviruses, including the spike glycoprotein (S) S1 subunit of SARS‐CoV‐2 in some IVIg preparations. Dalakas et al^53^ further expanded the study by examining several batches of commercially available IVIg products. In his study,^53^ even if produced before the SARS‐CoV‐2 pandemic, IVIg cross‐reacted against SARS‐CoV‐2 S1 antigen, in some cases the titres were like those detected in patients suffering from COVID‐19 infection. According to the Author,^53^ plasma from donors may contain antibodies which cross‐react against epitopes shared with ‘common cold’ coronavirus.

Indeed, in a study in uninfected individuals, Ng et al^54^ detected IgG antibodies against the S2 subunit of SARS‐CoV‐2, whereas COVID‐19 patients had high titres of Ig of all classes (IgG, IgA and IgM) directed against both the S1 and S2 subunits. In contrast, a recent study showed that current batches of IVIg lack cross‐neutralizing antibodies against SARS‐CoV‐2.^55^

The role of the antibodies cross‐reacting with SARS‐CoV‐2 is questioned. No data could prove that these antibodies, contained in IVIg preparations, have a protective role in SARS‐CoV‐2 uninfected individuals. These antibodies may directly act by exerting a priming effect on host immune response or play an immunomodulatory action on monocytes and tissue‐resident macrophages that are involved in the cytokines storm.^56^

Finally, SARS‐CoV‐2 may act as a superantigen to trigger the development of Multisystem Inflammatory Syndrome in Children (MIS‐C) as well as cytokines storm in adult COVID‐19 patients^22^, ^57^, ^58^, ^59^; the binding epitope on S subunit harbours a sequence motif unique to SARS‐CoV‐2 which is highly similar in both sequence and structure to another superantigen, the bacterial staphylococcal enterotoxin B (SEB). Due to structural similarities between SEB and the SARS‐CoV‐2 S subunit, it is possible that antibodies against SEB cross‐react with SARS‐CoV‐2 motif preventing in this way superantigen‐mediated T‐cell activation and cytokine release observed in COVID‐19^60^; this cross‐reactivity might be valid for IVIg, since it was demonstrated that IVIg contain neutralizing antibodies against SEB.^60^

## 5 IVIG IN COVID‐19 DISEASE

### 5.1 Analysis of published reports

The use of IVIg in COVID‐19 has been initially reported by Cao et al,^61^ who described its efficacy in three deteriorating patients. Subsequent reports described the benefit and safety of IVIg in critically ill patients COVID‐19.^62^, ^63^, ^64^, ^65^, ^66^, ^67^, ^68^, ^69^, ^70^, ^71^, ^72^, ^73^, ^74^, ^75^, ^76^, ^77^ Globally most of the published papers reported a good clinical response, confirmed by resolution of lung lesions with normalization of oxygen saturation and main laboratory parameters, and global improvement in clinical status.^62^, ^63^, ^64^, ^65^, ^66^, ^67^, ^68^, ^69^, ^70^, ^71^, ^72^, ^73^, ^74^, ^75^, ^76^, ^77^ Table 1 shows the main data of IVIg therapy in adult COVID‐19 disease. The heterogeneity of data collected does not allow a direct comparison. The different dose and duration of IVIg therapy, other concomitant treatments and the different clinical conditions of the patients make standardization difficult.

**TABLE 1:** Main studies on IVIg treatment in COVID‐19 disease

	IVIg dose and duration	Other therapies	Primary outcome in IVIg treated vs controls
Sakoulas et al,^69^ 2020 USA (open‐label RCT in 16 IVIg treated vs 17 controls)	0.5 g/kg/day for 3 days	Glucocorticoids, antivirals, convalescent plasma	Lower rate of progression to mechanical ventilation, shorter hospital and ICU stay, and greater improvement in PaO2/FiO2. ↓ ferritin and IL‐6
Xie et al,^63^ 2020 China (single centre retrospective in 58 IVIg treated)	20 g/day for 2‐5 days	Glucocorticoids, antiviral, antibiotic	If IVIg started <48 h of admission, improved pulmonary function, reduced days in hospital/ ICU and improved 28‐day mortality
Herth et al,^80^ 2020 USA and Germany (multicentre retrospective in 12 IVIg treated)	0.5‐2 g/kg/day in 1‐4 days	Glucocorticoids, antiviral, antibiotic, tocilizumab in 2 pts	If started <4 day of admission, improved pulmonary function reduced hospital and ICU stay
Shao et al,^62^ 2020 China (multicentre retrospective in 174 IVIg treated vs 151 controls)	0.1‐0.5 g/kg/day for 5‐15 days	Glucocorticoids, antivirals, antibiotics	Improved organ functions. If IVIg given ≤7 days at >15 g per day, reduced 60‐day mortality and ↓ CRP and ↓IL‐6
Gharebaghi et al,^74^ 2020 Iran (RCT in 30 IVIg treated vs 29 controls)	20 g/day for 3 days		Reduced mortality confirmed at the multivariate regression analysis
Zhou et al,^66^ 2020 China (single centre retrospective in 40 IVIg treated)	10‐20 g/day for 4‐26 days	Glucocorticoids, antivirals, antibiotics, interferon	Improved oxygenation index and pulmonary lesions with reduced mortality. ↓CRP and ↓ CK
Cao et al,^67^ 2021 China (multicentre retrospective study in 26 IVIg treated vs controls)	2 g/kg in 2‐5 days plus SoC <2 weeks of disease onset	Standard of care	Lower 28‐day mortality rate. Normalization of IL‐6, IL‐10 and ferritin
Esen et al,^70^ 2021 Turkey (single centre retrospective in 51 IVIg treated vs 42 controls)	30 g/day for 5 days	IVIg and /or SoC: glucocorticoids, hydroxychloroquine, antivirals, antibiotics, Tocilizumab or anakinra depending on inflammatory markers, vitamin C	Improved ICU survival in IVIg (61%) vs controls (38%) (68 vs 18 days). ↓CRP. No differences in procalcitonin, IL‐6 or D‐dimer
Zantah et al,^71^ 2020 USA (single centre retrospective in 51 IVIg treated vs 33 controls)	0.5 g/kg/day for 5 days	Glucocorticoids, Anakinra vs Tocilizumab	In both groups: improved clinical outcome, reduced days in ICU. ↓ ferritin and ↓ LDH in living pts
Tabarsi et al,^72^ 2021 Iran (RCT in 52 IVIg treated vs 32 controls)	0.4 g/kg/day for 3 days	Hydroxychloroquine, antivirals, supportive care	No differences in mortality rate and need of mechanical ventilation
Hou et al,^73^ 2021 China (single centre retrospective cohort study in 47 IVIg treated vs 66 controls)	0.5‐2 g/kg/day	Glucocorticoids	Reduced mortality and use of mechanical ventilation (25.5% vs 7.6%, *P* <.008)
Liu et al,^76^ 2021 China (multicentre retrospective study in severe and non‐severe patients (421 IVIg vs 429 controls)	Median IVIg dose 9.85 g/day for survivors and 10.42 g/day for non‐survivors with a median duration of 9.5 days for all patients	Glucocorticoids, antivirals	No differences in 28‐day mortality and Covid‐19‐related complications
Huang et al,^78^ 2021 China (retrospective study in non‐severe 45 IVIg treated vs 90 controls)	10 g/day for 3 days in 8 patients; 10 g/day for 5 days in 13 patients; 20 g/day for 3days in 16 patients; 20 g/day for 5 days in 8 patients	Glucocorticoids, hydroxychloroquine, lopinavir/ritonavir, Chinese medicine, thymosin α, arbidol	No benefit from IVIg vs SoC in mortality rate, progression to severe disease, duration of fever, virus clearance time, length of hospital stay, use of antibiotics
Raman et al,^79^ 2021 India (open‐label multicentre randomized study in non‐severe patients 47 IVIg treated vs 49 controls)	0.4 g/kg/day for 5 days plus SoC:	Antibiotics and lopinavir/ritonavir	Reduced use of mechanical ventilation, hospital and ICU stay. Reduced days to clinical improvement

In their large multicentre retrospective study conducted from December 2019 to March 2020, Shao et al^62^ experimented the use of IVIg in 174 patients (mean age 61 ys with 64% males) versus 151 controls (mean age 56 ys with 51% males). Less than 50% of patients had associated comorbidities, such as hypertension, diabetes, and coronary heart disease. Both groups were at a severe (68%) or critical (32%) stage of the disease and received standard of care (SoC) therapy based on glucocorticoids, antivirals and antibiotics. IVIg administered at a dose of 0.1‐0.5 g/kg/day for 5‐15 days lead to a significant decrease of 28‐day mortality in critical‐type patients in IVIg group, with no modifications on the duration of the hospital stay. No major benefit on survival was detected in severe‐type patients treated with IVIg as compared with those not treated with IVIg. Thus, according to this study,^62^ it seems that IVIg was more active in more critical patients with parallel reduction of Interleukin (IL)‐6 and C‐reactive protein (CRP). Even if patients showed different baseline characteristics among groups and there was some heterogeneity in treatments performed in the different centres, the major advantage of this study relies on the huge number of enrolled patients.

Other retrospective studies in severe and in critically ill patients confirmed these positive results. In their single‐centre study in 58 COVID‐19 patients (mean age 63 ys with 62% males) Xie et al^63^ reported a reduction in 28‐day mortality and ICU stay with improved pulmonary function. Major benefit followed IVIg administration at the dose of 20 g/days within 48 hours from admission. Even in this series, patients received IVIg associated with glucocorticoids, antivirals and antibiotics. Unfortunately, in this study, there was no control group and results were not stratified according to the severity of the disease at study entry.^63^

The benefit of IVIg in critically ill patients was further confirmed in several studies reported in different Countries, including USA, Iran, Italy, China, Turkey and Bhutan.^64^, ^65^, ^66^, ^67^, ^68^, ^69^, ^70^ In a large single‐centre retrospective study, Esen et al^70^ documented a striking improvement in overall ICU survival in 51 severe patients treated with IVIg as compared to 42 controls (61% of surviving patients vs 38%; odds ratio: 2.2, 95% confidence interval: 0.9‐5.4, p 0.091 after controlling for baselines imbalances) with a significant increased median survival (68 vs 18 days, *P* = 0.014). In this series patients and controls received a complex combined treatment comprising methylprednisolone (200 mg/day), hydroxychloroquine (HCQ, 800 mg/day loading dose, and 400 mg/day as maintenance dose for 5 days), antivirals and azithromycin. Intervention group received 5% IVIg at the dose of 30 g/day for 5 consecutive days. According to the levels of inflammatory markers, tocilizumab or anakinra were added to the above schedule in <5% of cases in both groups.^70^ As in other reports, however, the baseline disease severity was not perfectly balanced between intervention and control groups, being this latter group characterized by a slightly more severe disease.

Another study explored the role of IVIg in critically ill patients as add‐on treatment with Tocilizumab or Anakinra. In their single‐centre retrospective study, Zantah et al^71^ documented improved clinical outcomes and reduced stay in ICU in 51 patients (mean age 62 years, 65% M) as compared with 33 controls (mean age 57 years, 61% M). The clinical benefit was mirrored by a reduction in ferritin and LDH in living patients. Even if retrospective and without a control group, this study is interesting since it compares the association of Anakinra and IVIg versus Tocilizumab in 84 consecutive patients (51 in the Anakinra/ IVIg group and 33 in the Tocilizumab group). Both intervention arms had positive results with improved clinical outcomes.^71^

A randomized controlled trial conducted by Tabarsi et al^72^ compared 52 critically ill patients to 32 controls. Both IVIg treated patients (at a dose of 0.4 g/kg/day for 3 days) and those in the control group received hydroxychloroquine and lopinavir/ritonavir. Despite early IVIg administration (mean time 3.84 ± 3.35 days after the admission), the study did not detect any significant difference between the two groups regarding the use of mechanical ventilation and mortality rate.^72^ However, the time interval between admission and IVIg administration significantly correlated with the hospital and ICU stay (*P* = 0.01 and <0.001, respectively) in surviving patients.^72^ Even if it is difficult to draw definite conclusions, Authors documented a reduction in hospital and ICU stay as soon as IVIg treatment starts.^72^ A recent single‐centre retrospective cohort study revised the use of IVIg in 47 critically ill COVID‐19 patients. The patients with the most severe disease received IVIg, but no dose has been reported. According to Hou et al^73^ more patients in the IVIg group reached the primary outcome of the study with reduced mortality and use of mechanical ventilation compared with control group (25.5% vs 7.6%, *P* < 0.008).

In patients with less critical disease, Sakoulas et al^69^ reported the preliminary results of a prospective randomized open‐label trial comparing IVIg (0.5g/kg/day for 3 days) plus methylprednisolone (40 mg 30 minutes before IVIg) versus SoC. Age and sex were matched among the groups (16 patients in IVIg with median age 58 ys with 63% males; and 17 in SoC group with median age 51 ys with 59% males). According to their results, IVIg significantly improved oxygen saturation (*P* < 0.01, Mann‐Whitney *U* test), the need for mechanical ventilation (*P* < 0.038, Fisher exact test), with concomitant reduction of median ICU and hospital stay (*P* = 0.006 and *P* = 0.01, Mann‐Whitney *U* test respectively).^69^

In a recent randomized placebo‐controlled double‐blind clinical trial, Gharebaghi et al^74^ demonstrated the benefit of IVIg in severe patients with Covid‐19. In treatment group, 30 patients with refractoriness to initial treatments received IVIg (20g for three consecutive days) plus SoC, and were compared to 29 patients receiving only SoC. This study clearly documented a shorter mortality rate in IVIg treated patients (6 [20.0%] vs 14 [48.3%] respectively; *P* = 0.025). This data were further confirmed at the multivariate regression analysis showing IVIg treatment had a significant impact on mortality rate (aOR = 0.003 [95% CI: 0.001‐0.815]; *P* = 0.042) and is an independently associated factor of mortality.^74^

Omma A. et al^75^ reported their single‐centre experience about administration of IVIg in 46 patients with severe diseases, characterized by refractoriness to antivirals or anti‐inflammatory agents and/or with associated comorbidities. Mortality was directly proportional to disease's severity and higher in patients with COVID‐19 related complications (such as myocarditis, adult multisystem inflammatory syndrome, haemophagocytic lymphohystiocytosis like syndrome), with a survival rate of 33.7% in refractory patients treated with IVIg. At the opposite, Liu et al^76^ found that IVIg treatment in severe patients did not significantly reduce 28‐day mortality (ATE = 0.008, 95% CI −0.081‐0.097, *P* = 0.863) or the incidence of COVID‐19‐related complications in 406 patients enrolled per group (IVIg vs non‐IVIg treated patients).

Data on mortality are thus non‐conclusive. However, several studies reported a reduced mortality in critically ill patients,^62^, ^63^, ^73^, ^74^, ^75^ and a meta‐analysis performed by Xiang et al^77^ confirmed the clinical efficacy of IVIg on critically ill patients with reduced mortality as compared with controls [RR = 0.57].

Few studies explored the use of IVIg in mild COVID‐19. In a retrospective study, Huang et al^78^ described 45 non‐severe patients vs 90 controls. Doses and duration of IVIg administrations were variable. They demonstrated that in non‐severe patients there was no benefit in term of duration of fever, virus clearance time, length of hospital stay, use of antibiotics, progression to severe disease (control vs IVIG group 3.3% vs 6.6%, *P* = 0.376) or mortality (0% vs 2.2%, *P* = 0.156).^78^ In their open‐label multicentre randomized study in non‐severe patients, Raman et al^79^ documented the benefit of IVIg (at the dose of 0.4 g/kg for 5 days) with improvement in the need of mechanical ventilation and reduced days spent in ICU and/or hospital. Patients treated with IVIg had a shorted period to clinical improvement as compared with those treated only with SoC.

Some interesting issues have been raised in these studies. As reported in different studies the timing and dosage of IVIg can have a crucial role. First, it seems that an early administration of IVIg is relevant for a successful outcome. Even if IVIg has been reported beneficial in patients with long‐standing COVID‐19,^80^ the infusion within 3^80^ or 7 days from hospitalization^62^ or within 48 h from ICU admission^63^ improve the final prognosis. This issue is related to the timing of administration of the drugs in COVID‐19 patients.^81^ The difficulties in treating this new disease are due that we do not know the exact stages of the viral infection, neither the different steps of the immune response to the virus. Since a sequence of different phases characterize the SARS‐CoV‐2 infection, it is important to give the right drug at the right point. In this regard, it seems that the major advantages of IVIg are linked to the infusion at early stages of the infections which lead to improved survival.^63^, ^69^, ^80^

Second, even the IVIg dose can influence the outcome. The schedules of IVIg administration varied greatly among the studies. In most of the papers, IVIg treatment was based on the administration of a dose ranging from 0.3 to 0.6 g/kg/day for 3 or 5 days.^63^, ^65^, ^69^ Many single cases reported quite similar high‐dose regimens of 20 to 30 g/day delivered over 4 to 6 days.^61^, ^64^According to Shao et al,^62^ the benefit of IVIg, as reflected by a significant reduction in 60‐day mortality, came from the use of high‐dose IVIg, more than 15 g per day, the same dosage linked to anti‐inflammatory properties of IVIg.^2^

Third, among the advantages of IVIg treatment is the rapid response in patients as documented by Herth et al^80^ and further confirmed by others.^70^, ^75^

Fourth, in almost all studies, IVIg was well tolerated.^67^, ^82^, ^83^ Cao et al^67^ reported the safety profile of IVIg in their series of 26 COVID‐19 patients treated with high‐dose IVIg with final improvement in 28‐day mortality. They did not detect major side effects, three patients (5.9%) reported self‐limiting palpitation (n = 1), dizziness (n = 1) and rash (n = 1). Finally, the concern of the thrombotic risk has not been confirmed, even in subjects with high D‐dimers levels.^69^ It is even possible that IVIg interferes and lessen the risk of thrombotic events.

IVIg treatment retains a good safety profile coming from a large use during the years,^83^ whereas clinicians could be less familiar with other kinds of treatment such as anti‐IL‐6 or new antivirals. Another advantage is the use in Countries with low‐income or no access to other more expensive drugs.^82^

### 5.2 Limits of the published reports

Despite these encouraging results, it should be reminded that most of the studies are retrospective and single centre‐based and describe series with a small number of patients, which can reduce the power and the strength of the positive findings. Only few employed a control group.^62^, ^67^, ^69^, ^70^, ^71^, ^72^, ^74^ Clinical conditions of patients varied among the studies with inclusion of both moderate and severe patients, in some reports without analysing the results in specific subgroups of patients.^69^ Some of the clinical outcomes varied among studies, such as ventilation timing of the start, ICU and hospital discharge and overall survival (analysed at different points). Not all the studies reported the impact of IVIg on the same laboratory parameters, including CRP, IL‐6 or ferritin. As discussed before, IVIg timing and dosage are different in different studies. Finally, the concurrent use of glucocorticoids or drugs that can be effective on COVID‐19, such as antivirals, tocilizumab or anakinra, could impact on the analyses of the results in some studies. Another important issue is that related to the shortage of blood products, including IVIg, due to the marker reduction in blood donations, linked to the pandemic.

Yet the overall conclusion favours the use of IVIg, with resolution of lung lesions and return to normal oxygen saturation, concomitant reduction of the main laboratory parameters with subsequent decrease in mortality in critically ill or severe patients with COVID‐19 disease.

## 6 SARS‐COV‐2 AS AN AUTOIMMUNE VIRUS

As widely reported in literature, IVIg has a beneficial effect in several and distinct autoimmune disorders.^84^, ^85^, ^86^ In recent years, conventional (SCIg) and facilitated (fSCIg) subcutaneous immunoglobulin has been proposed as another therapeutic option to restore immune system imbalance in selected autoimmune diseases such as inflammatory myopathies.^87^, ^88^, ^89^, ^90^ Since recent studies explore the hypothesis that SARS‐CoV‐2 may act as a trigger in the development of autoimmune or autoinflammatory disorders, another interesting use of IVIg is linked to the positive results obtained in several autoimmune diseases induced by SARS‐CoV‐2.^91^, ^92^, ^93^

Holding the widest single‐strand RNA among organisms, Coronaviruses’ transcriptome retains a vast ability to interact with our defence system. The interaction could also be favoured by a molecular similarity between viral and some human peptides, whose dysfunction can trigger autoimmune diseases.^93^, ^94^, ^95^ Its sporadic transcription and recombination give rise to a complex number of epitopes contributing to autoimmune mechanisms as molecular mimicry, bystander activation, epitope spreading and cytokines storm.^92^ The literature suggests that this dysregulation in genetically predisposed individuals not only leads to development of interstitial pneumonia and respiratory failure, the main COVID‐19 disease features, but also to autoimmune diseases.^91^

Giving support to the previous assumptions, in recent papers multiple diseases related to SARS‐CoV‐2 infection have emerged, all of which can be reported to other microorganisms except for one SARS‐CoV‐2 specific disease, a form of Kawasaki‐like multisystem inflammatory syndrome in adults.^92^ Table 2 shows the autoimmune diseases elicited by SARS‐CoV‐2 and also by other microorganisms. Additional manifestations of COVID‐19 infection are antiphospholipid antibodies mainly without thrombosis,^91^ arthralgia with/without myalgia, thrombocytopenia, Raynaud's phenomenon, psoriasis flares, crescentic glomerulonephritis, collapsing glomerulopathy, ulcerative colitis and inflammatory bowel disease flares associated to SARS‐CoV‐2 infection, many on the list resulted responding to standard immunomodulatory drugs.^92^

**TABLE 2:** Autoimmune diseases reported in COVID‐19

Systemic autoimmune diseases	
New onset	Systemic Lupus Erythematosus^92^, ^93^
	Arthritis (not infectious)^92^
	Systemic vasculitis^92^
	Antiphospholipid antibodies^92^
	Kawasaki disease^91^, ^93^
	Multisystem Inflammatory Syndrome in Children (MIS‐C)^91^, ^92^, ^93^, ^94^, ^95^, ^96^, ^97^, ^98^, ^99^, ^100^, ^101^, ^102^, ^103^, ^104^, ^105^
Exacerbation	Antiphospholipid Syndrome^92^
	Systemic Lupus Erythematosus^92^
	Rheumatoid Arthritis^92^
Organ specific autoimmune diseases	
New onset	Immune thrombocytopenic purpura^92^, ^93^
	Autoimmune idiopathic haemolytic anaemia^92^
	Evans syndrome^92^, ^111^
	Pemphigus vulgaris^112^
	Guillain‐Barré syndrome^91^, ^92^, ^93^
	Miler‐Fisher Syndrome^91^, ^92^, ^93^
	Acute disseminated encephalomyelitis^92^, ^93^, ^94^, ^95^, ^96^, ^97^, ^98^, ^99^, ^100^, ^101^, ^102^, ^103^, ^104^, ^105^, ^106^, ^107^, ^108^, ^109^, ^110^, ^111^, ^112^, ^113^, ^114^, ^115^
	Devic syndrome^92^
	Goodpasture's syndrome^92^
Exacerbation	Myasthenia gravis^92^, ^93^, ^94^, ^95^, ^96^, ^97^, ^98^, ^99^, ^100^, ^101^, ^102^, ^103^, ^104^, ^105^, ^106^, ^107^, ^108^, ^109^, ^110^, ^111^, ^112^, ^113^
	Multiple sclerosis^92^

At this regard, the work of Zhou et al^96^ describes 21 patients who developed autoimmune disease‐related autoantibodies to anti‒SSA/Ro antibody in 45% and antinuclear antibody in 50% of cases, all benefiting from immunosuppressive therapy. Other autoantibodies reported in literature are anti‐nuclear antibodies (ANA), antiphospholipid antibodies (aPls) as anti‐cardiolipin (aCL) and anti‐β2 glycoprotein 1 (aβ2GP1), anti‐IFN antibodies, anti‐MDA5 antibodies, LAC Lupus anticoagulant, pANCA, cANCA, anti‐CCP antibodies^2^ and the recently described anti‐Angiotensin‐Converting Enzyme 2 (ACE2).^97^ McMillan et al^97^ exposes how SARS‐CoV‐2 autoimmunity hypothesis goes in agreement with other autoimmune disorders, relating to pathogenesis and symptoms. Anti‐ACE‐2 autoantibodies cover the role of the primary effector, followed by the antibodies listed above. Anti‐IFN antibodies seem related to a most severe course and are described to act thanks to bystander effect.^97^

The theory of autoimmunity elicited by SARS‐CoV‐2 has been reinforced by the recent RECOVERY Trial^98^ showing the benefit of the steroid dexamethasone for immunosuppression. Additionally, high‐dose methylprednisolone has also been proposed as a rescue, second‐line treatment for patients who did not respond well to other therapies.^99^

## 7 IVIG IN SARS‐COV‐2 INDUCED AUTOIMMUNE DISEASES

Revising the literature on COVID‐related autoimmune diseases treated with IVIg, we found some papers describing Guillain‐Barré syndrome (GBS) and his different subtypes. Assini et al^100^ reported two cases of COVID‐related polyradiculopathy. Although both patients presented atypical GBS features (the first was GBS overlapping with Miller Fisher's syndrome, the second was associated with severe autonomic neuropathy), neurological symptoms responded well to the administration of high‐dose IVIg (0.4 g/kg/day for 5 days). Farzi et al^101^ detailed another case of GBS in COVID‐19 affected patient, in which neurological symptoms appearing 10 days after pneumonia resolved after IVIg treatment (0.4 g/kg/day for 5 days) with the recovery of strength and ability to walk. In his article, Dalakas^102^ discussed 11 cases of GBS related to SARS‐CoV‐2 infection. In one patient, polyneuroradiculopathy was the first symptom of the disease, in the others, followed respiratory and systemic symptoms. Almost all patients (one died) benefited from high‐dose IVIg therapy. Antiganglioside antibodies were tested in five patients. Only one with Miller Fisher's syndrome was positive for GD1b ganglioside antibodies, different from the more typical GQ1b.^103^ This finding led to an interesting consideration: viral spike protein binds, in addition to the ACE‐2 receptor, also glycoproteins and gangliosides that contain sialic acid residues.^103^ A possible cross‐reactivity between epitopes within gangliosides bound by spike protein and glycolipids surface sugars of peripheral nerves could explain the autoimmunity.^102^ Although the autoimmune pathogenic basis is not completely known, all COVID‐19 related GBS described have responded to immunomodulatory therapy with IVIg.

The only COVID‐19 related auto‐inflammatory disease that appears to be a new entity is the hyper‐inflammatory Kawasaki‐like syndrome. In May 2020, the Centres for Disease Control and Prevention issued the definition for MIS‐C,^57^ the hyper‐inflammatory shock syndrome described during COVID‐19 pandemic.^58^ This new condition was initially associated with Kawasaki disease for both pathogenesis and some clinical features. Jones et al^105^ reported the first Kawasaki disease (KD) COVID‐19 related in a 6‐month‐old infant with mild respiratory symptoms. The patient was treated with a single dose of IVIg (2g/ kg) and low‐dose aspirin.^105^ In April 2020 Verdoni et al^106^ found a high incidence of Kawasaki‐like disease in Bergamo province in Italy during the first COVID‐19 pandemic months. They compared paediatric patients with KD from 2015 to January 2020 (group 1), and the new cases of Kawasaki‐like disease arose between March 2020 and April 2020 (group 2). In group 2 the average age and the incidence were higher than in group 1. Moreover, in group 2, 50% of patients did not have the criteria for complete KD and the disease was clinically more severe with respiratory, gastrointestinal and cardiovascular involvement and meningeal signs. Half of the patients in group 2 (vs 0 in group1) developed macrophage activation syndrome (MAS) and Kawasaki disease shock syndrome, and 60% (vs 10%) had echocardiographic abnormalities. The biochemical tests showed significant lymphopenia and thrombocytopenia in group 2. As for therapies, all patients were treated with IVIg 2g/kg in single dose, but 80% in group 2 also required steroid treatment (vs 16% in group 1). All responded to the treatment. In group2, eight patients had positive IgG/IgM tests for SARS‐CoV‐2, only two positive swab tests.^106^

Chiotos et al^107^ reported six cases of MIS‐C. SARS‐CoV‐2 IgG test was positive in five, in one it was not performed. Clinically all presented with fever and shock, four diarrhoea, five abdominal pain/emesis, one conjunctivitis, four respiratory failure, four neurological symptoms (headache, altered mental status, irritability and neck rigidity). At the initial echocardiography, only one had coronary dilations, but four had reduced left ventricular function. Five patients required vasoactive amine support, and all were treated with at least one infusion of IVIg (2g/ kg), two received a second dose of IVIg, five received methylprednisolone 2 mg/kg/day (two patients started with 30 mg/kg/day for 3 days).^107^

Pouletty et al^108^ compared, in a multi‐centre retrospective cohort study, new MIS‐C cases in Paris region since April 2020, with a historical cohort of ‘classic’ KD patients. All patients had a positive test (serological or swab test) for SARS‐CoV‐2 and/or close contact with a positive COVID‐19 individual. Among MIS‐C patients, 15 (94%) received IVIg (2g/ kg) and nine needed a second line of treatment: four received a second IVIg infusion; the others received steroids or biologics. All 15 patients also received aspirin at anti‐inflammatory or antiplatelet dose. A patient who presented with typical KD did not receive treatment. The average age of MIS‐C patients was 10 years, higher than the classic KD patients. There was also a higher incidence of myocarditis, pericarditis, gastrointestinal symptoms, as well as organ failure related to the cytokines storm.

MIS‐C is a new clinical condition with some features in common with KD.^58^ As it happens for KD, IVIg is considered the first line of treatment, but additional therapeutic options, such as steroid therapy or a second infusion of IVIg, seem to be necessary in severe or complicated cases (MAS or shock).

The development of autoantibodies is not always directly related to the development of autoimmune disease, which can occur after years. Raahimi et al^109^ described the case of a patient who developed acute inflammatory demyelinating polyneuropathy 53 days after having COVID 19 pneumonia. Again, treatment with standard dose IVIg (2 g/kg over 5 days) was effective: neurological symptoms improved and the patient recovered gradually. Table 3 shows other COVID‐related autoimmune disease treated with IVIg.^109^, ^110^, ^111^, ^112^, ^113^, ^114^, ^115^

**TABLE 3:** COVID‐related autoimmune diseases treated with IVIg. In pemphigus vulgaris^111^ and myasthenia gravis exacerbation,^112^ IVIg was preferred to other first‐line immunosuppressive therapies in order not to increase the infectious risk

Disease (Ref.)	Treatment and dose
Guillain‐Barré syndrome^91^, ^92^, ^93^	IVIg 0.4 g/kg/day for 5 days
Kawasaki disease^91^, ^93^	IVIg 2 g/kg single dose +aspirine
Multisystem Inflammatory Syndrome in Children (MIS‐C)^91^, ^92^, ^93^, ^94^, ^95^, ^96^, ^97^, ^98^, ^99^, ^100^, ^101^, ^102^, ^103^, ^104^, ^105^	IVIg 2 g/kg single or double dose +high‐dose steroid +aspirine
Evans syndrome^111^	IVIg 1 g/kg/day +high‐dose steroids +plasmapheresis
Pemphigus vulgaris^112^	IVIg only as first line or associated with high‐dose steroid
Myasthenia gravis exacerbation^113^	IVIg 2 g/kg in 5 days as first line
Immune thrombocytopenic purpura^114^	IVIg 1 g/kg +high‐dose steroid as first line
Acute disseminated encephalomyelitis (ADEM)^115^	IVIg 2 g/kg in 5 days +high‐dose steroid

Finally, it is important to pay attention to the post‐COVID (ie short‐term) and long‐COVID (ie long‐term) manifestations appearing in people recovering from the SARS‐CoV‐2 infection. Any organ system can be involved, in particular immune system. Among the sequelae of COVID‐19, a Multisystem Inflammatory Syndrome has been described in adults, called MAS‐A.^116^

Although only single cases have been described, the development of autoimmune diseases in COVID‐19 patients may also occur long after resolution of the infection. In this way the ‘Post‐COVID syndrome’,^110^ which until now is not well defined and whose description closely resembles the post‐ICU syndrome, could therefore include autoimmune manifestations. Longitudinal observational studies will be fundamental to better define the ‘Post‐COVID syndrome’ and the weight of immunological disorders in its pathogenesis.

## 8 SARS‐COV‐2 VACCINE INDUCED AUTOIMMUNE DISEASES

In the least months, some papers reported the occurrence of autoimmune diseases following SARS‐CoV‐2 vaccination. Shemer et al^117^ was the first to describe nine cases of a new acute‐onset facial nerve palsy appearing after the administration of the BNT162b2 vaccine. Other reports described the new appearance or in some case the relapses of a pre‐existing autoimmune disease (17 flares and 10 new onset autoimmune diseases, ITP, GBS, and autoimmune hepatitis) after different type of SARS‐CoV‐2 vaccine.^118^, ^119^, ^120^, ^121^, ^122^ Even if the precise mechanisms of vaccine‐induced autoimmune manifestation or disease are not completely understood, it is possible that the vaccine behaves as a trigger in predisposed patients. Two papers^121^, ^122^ reported the successful response to IVIg in ITP and GBS. Graf et al^123^ reported the case of a patient who developed immune thrombotic thrombocytopenia after vaccination against SARS‐CoV‐2 adenoviral vector vaccine (VITT: Vaccine Induced Thrombotic Thrombocytopenia) successfully treated with high dose IVIg and anticoagulation.

## 9 CONCLUSIONS

Despite the presence of clinical heterogeneity and some methodological concerns, a global concordance was detected among the main studies: high‐dose IVIg (>15 −20 g/day) at an early start of infection could positively impact on the overall prognosis of COVID‐19 patients. Moreover, the use of IVIg can improve the survival of the patients, especially in those with lymphocytopenia, as commonly occurring in COVID‐19.^124^

The IVIg treatment cannot resolve completely the clinical condition, still can contribute to attenuate the burden of the disease, reducing the stay in ICU and the demand for mechanical ventilation. This is very important, since it can help to reduce the health resources consumption and thus the economic impact of the severe cases during the pandemic.

## CONFLICT OF INTEREST

The authors declare that they have no competing interests.

## AUTHOR CONTRIBUTIONS

MGD, YS: conception of the work, intellectual production, writing of the manuscript and critical revision. MGD, MAP, AP, EL, CM, GM: revision of the literature, drafting text and tables. EL: supplying the figure. All: approval of the final version to be published the manuscript. All: Agreed to be accountable for all aspects of the work in ensuring that questions related to the accuracy or integrity of any part of the work are appropriately investigated and resolved.
